# Prevalence and risk factors of hepatitis B and C virus infections among the general population and blood donors in Morocco

**DOI:** 10.1186/1471-2458-13-50

**Published:** 2013-01-18

**Authors:** Warda Baha, Abderrahim Foullous, Noureddine Dersi, Thierry Paluku They-they, Khadija El alaoui, Nadia Nourichafi, Bouchra Oukkache, Fatiha Lazar, Soumaya Benjelloun, My Mustapha Ennaji, Abdelouhad Elmalki, Hassan Mifdal, Abdelouaheb Bennani

**Affiliations:** 1 Molecular Biology Laboratory, Department of Medical Biology, Pasteur Institute of Morocco, 1, Place Louis Pasteur, Casablanca, 20360, Morocco; 2 Virology, Hygien & Microbiology Laboratory, Faculty of Science and Technology, 146, route de Rabat, Mohammedia, 20650, Morocco; 3 Regional Blood Transfusion Center, 1, rue Faidouzi Mohamed, Casablanca, 20360, Morocco; 4 Hematology Laboratory, Centre Hospitalier Universitaire Ibn Rochd, 1, Rue des Hôpitaux, Casablanca, Morocco

**Keywords:** Hepatitis B, Hepatitis C, Prevalence, General population, Risk factors, Blood donors, Morocco

## Abstract

**Background:**

Viral hepatitis is a serious public health problem affecting billions of people globally. Limited information is available on this issue in Morocco. This cross-sectional study was undertaken with the aim of determining the seroprevalence and risk factors of hepatitis B virus (HBV) and hepatitis C virus (HCV) among the general population and among blood donors.

**Methods:**

Blood samples from volunteers, have been screened with ELISA tests for detecting the hepatitis-B surface antigen (HBsAg) and anti-HCV. Within the seroreactive patients for HCV in the general population, RT-PCR was performed by the Cobas Ampliprep/Cobas Amplicor.

**Results:**

HCV and HBV-seropositivity was documented in 1.58% and 1.81% out of 41269 and 23578 participants respectively from the general population. Two patients were found to be co-infected. HCV-RNA was detected by PCR in 70.9% of the 195 anti-HCV positive subjects. The anti-HCV prevalence was not different among males and females (P = 0.3). It increased with age; the highest prevalence was observed among subjects with >50 years old (3.12%). Various risk factors for acquiring HCV infection were identified; age, dental treatment, use of glass syringes and surgical history. In addition to these factors, gender and sexual risk behaviors were found to be associated with higher prevalence of hepatitis B. The HBV positivity was significantly higher among males than females participants in all age groups (P < 0.01). The peak was noticed among males aged 30–49 years (2.4%). None of the 152 persons younger than 20 years had HBsAg or anti-HCV. The prevalence of anti-HCV and HBsAg among 169605 blood donors was 0.62% and 0.96% respectively.

**Conclusions:**

Our study provided much important information concerning hepatitis B and C prevalence and risk factors; it confirmed the intermediate endemicity for HCV infection and pointed to a decreasing trend of HBV incidence, which might reclassify Morocco in low HBV endemicity area. This could be attributed primarily to the universal HBV vaccination among infants and healthcare workers over the past 13 years. HCV and HBV infections in the present survey were mainly associated with nosocomial exposures. Prevention and control of HBV infection are needed to reduce HBV transmission between adults.

## Background

Hepatitis B virus (HBV) and hepatitis C virus (HCV) are among the principal causes of severe liver disease, including hepatocellular carcinoma (HCC) and cirrhosis-related end-stage liver disease. The World Health Organization (WHO) has estimated that there are 360 million chronically HBV infected people and 5.7 million HBV-related cases worldwide [1]. HBV is highly infectious and transmitted mainly via blood, body-fluid contact, and vertical transmission [2]. The hepatitis B surface antigen (HBsAg) in serum is the first seromarker to indicate active HBV infection, either acute or chronic [3]. A hepatitis B vaccine, available since 1982, has a high efficacy in the prevention of HBV transmission [2] and has brought about remarkable changes in the global epidemiology of HBV infection. HCV infection is also common worldwide. It is transmitted in a manner similar to HBV and it is estimated that about 3% of the world’s population carry HCV, with 3 to 4 million new infections per year [4]. Hepatitis C is generally asymptomatic, with a strong tendency (up to 80%) for progression to persistent infection [5]. Chronic HCV infection progresses at a variable rate to cirrhosis in 15 to 20% of patients, who then have a 1 to 4% annual risk of developing hepatocellular carcinoma in 20–30 years [6].

Morocco has been placed by the WHO into the intermediate zone of prevalence for both hepatitis B and C; however the exact number of persons infected is unknown for the reason that no large-scale epidemic data exist to assess the true HCV and HBV infection burden in Morocco. Although a few studies have been carried out, they were affected by a selection bias because most studies are generally based on subjects from risk groups or blood donors with lacking information on children and senior citizens not generally included in as specialized groups [7-9].

The scarcity of data and the degree to which they are out of date were the main reasons behind a nationwide cross-sectional survey aimed to determine the prevalence and risk factors of HCV and HBV infections in the general population and among blood donors to unravel the true status of viral hepatitis in apparently healthy Moroccan persons and thus provide valuable information about surveillance and estimation.

## Methods

### Study design, setting and population

#### General population

This was a nationwide cross-sectional survey enrolling in the large screening program for hepatitis B and C conducted by the Pasteur Institute of Morocco and carried out in eleven major Moroccan regions between December 2005 and June 2011. The target subjects in this study were the apparently healthy Moroccan individuals which were selected to be as representative of the demographic characteristics as possible in regarding areas of Morocco. To construct the national probability sample, a stratified, random cluster sampling method was used to select the study population. In the first stage, names of private and public organizations (companies, agencies, banks, factories, schools…) from different prefectures and provinces of regions were obtained using directories. Lists were compiled using excel tool to select 288 organisms. To invite the selected structures to participate in the survey, a letter was sent with standardized information regarding the study. Each organism has agreed to participate provided a list of the active or retired personnel (also students in case of schools). The study population was selected by a systematic 1:3 sampling procedure; every third person from each list was selected and approached for participation. The study was also open for all persons who wish to participate.

The investigative team performing the fieldwork consisted of physicians who performed clinical examination, paramedics who collected blood samples, and trained social workers who explain the objectives of the study and interview each participant. No willful participation was allowed and if any eligible candidate declined to participate, his or her name was dropped from the list of participants. An informed consent was obtained from the participating subjects and, in the case of the minors, the parents or tutors. A standard and individual questionnaire was used to gather data related to demographic and socio-economic characteristics, age, gender, present and past health status, history of jaundice, and possible risk factors for transmitting viral hepatitis.

In the end, 280 structures (97.3%) and 41 269 out of 41 311 participants (99.9%) agreed to participate in the survey. The first 17 691 persons recruited were tested only for anti-HCV Ab and the subsequent 23 578 persons were tested for both anti-HCV and HBsAg. All subjects were grouped by sex and age range. The collected blood was transferred to the Molecular Biology Laboratory at Pasteur Institute of Morocco under sterile conditions in ice bags, separated and stored at −70°C until laboratory testing was performed.

The HBsAg and the IgG antibodies to HCV markers were serologically assessed using a third generation enzyme immunoassay (Murex HBsAg Version 3, Abbott-Murex, South Africa) and (Murex, anti-HCV version 4.0 Abbott Kyalami, South Africa) respectively. Positive samples were retested for confirmation using an automated microparticle enzyme immunoassay (Abbott, AxSYM. System, Wiesbaden, Germany).

Anti-HCV–positive subjects were recalled for further evaluation at a subsequent date. A detailed clinical examination was performed and blood samples were further assessed for HCV RNA by polymerase chain reaction using the Cobas Ampliprep/Cobas Amplicor HCV Test (Roche diagnostics, Mannheim, Germany), (limit of detection 50 IU/ml).

#### Blood donors

The participants in this cross-sectional study were all volunteer blood donors who donated blood at the Casablanca Regional Blood Transfusion Center between January 1st, 2008 and December 31, 2010.

The donors were interviewed by physicians before donation; each donor was requested to fill a blood donor’s form and was obliged to satisfy the center’s criteria: age over 18 and less than 65, body weight over 50 kg, normal body temperature, absence of clinical signs of an acute infective response, no history of infectious and chronic diseases and information about any high-risk behavior. Therefore all participants selected in this study are presumed without a history of known or obvious risk factor. Care was also taken to make sure that no donor was used more than once in this study. Unsuitable donors were excluded.

All blood samples were routinely screened for transfusion-transmitted diseases (HBV, HCV, HIV and Syphilis) according to established screening procedures and tests; hence specimens were tested for HBsAg and anti-HCV antibodies by Murex HBsAg Version 3 and Murex Ag/Ab HCV Combination Assay (Abbott-Murex, South Africa) respectively. Reactive samples were retested in duplicate. Repeatedly reactive samples were confirmed by means of recombinant immunoblot assay for HCV (RIBA, Innogenetics). Samples were considered to be positive if were reactive for both tests.

All procedures were followed in accordance with the current revision of the declaration of Helsinki, approved by the ethical committee of Pasteur Institute of Morocco.

### Statistical analysis

Statistical analyses were performed by using SPSS 16.0 software. The overall prevalence of HCV and HBV markers was expressed as the percentage of seropositive samples. The prevalence odds ratios (OR) and Confidence intervals (CI) of 95% for OR estimates were also estimated. A level of p < 0.05 was used to indicate statistical significance.

## Results

### General population

In all, 41 269 (sex-ratio (M/F) = 2.30) serum samples were tested for anti-HCV Ab analysis and 23 578 (sex-ratio (M/F) = 1.84) subjects were enrolled for HBsAg detection. The overall mean age was 45 (± 10.9) years, ranging from 5 to 84 years.

**Figure 1:**
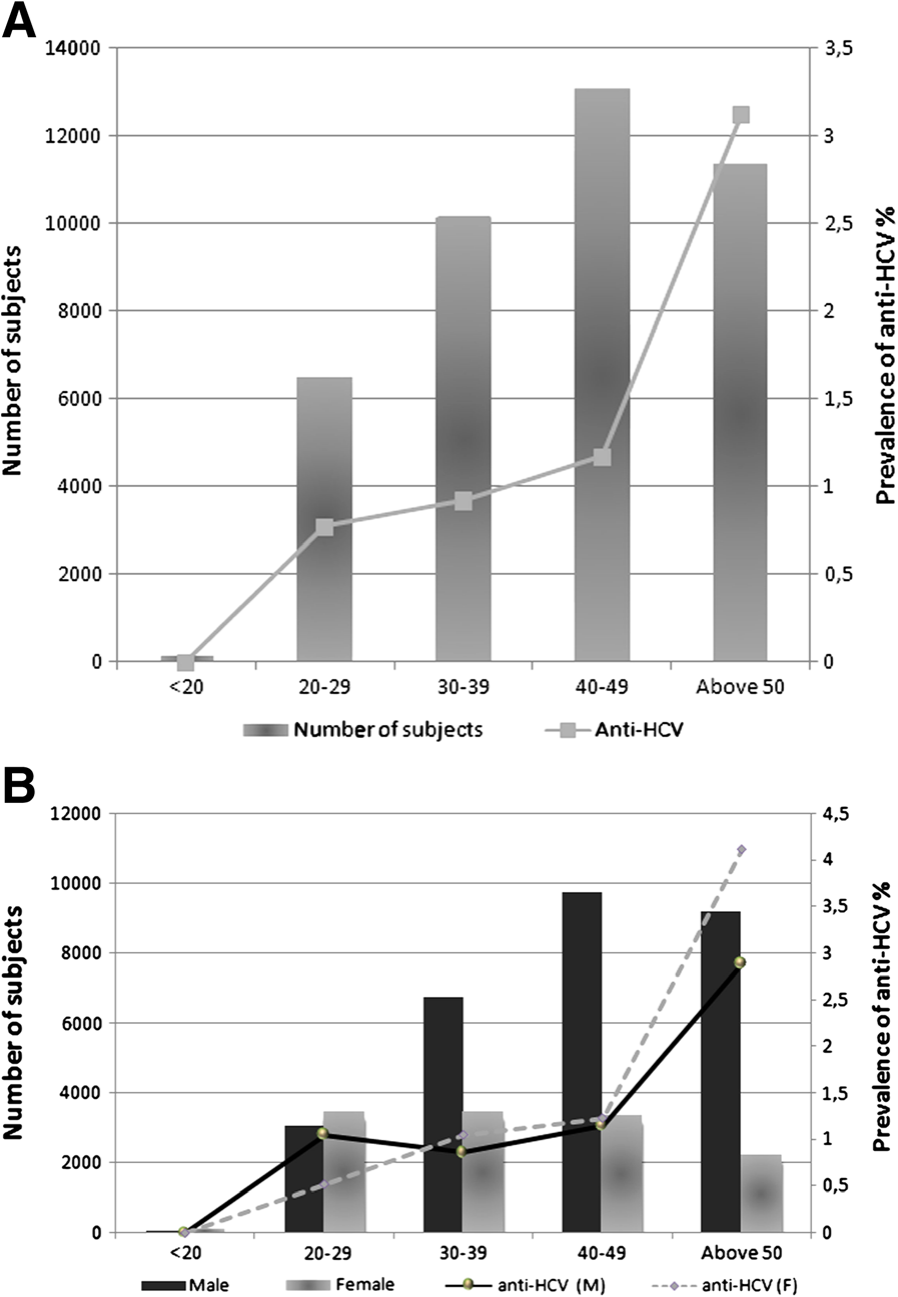
**Serological features of anti-HCV prevalence in Morocco.** Bars represent the number of subjects in each group indicated by left vertical axis, and the points represent the prevalence in each group indicated by right vertical axis. The horizontal axis represents the age ranges. **A**, Overall characterization of anti-HCV by age group. **B**, Overall characterization of anti-HCV by gender, M, male, F, female.

The seroprevalence of anti-HCV Ab was found to be 1.58%, corresponding to 651 seropositive patients out of 41 269 participants, of those individuals, viral RNA was detected in 70.9%. The prevalence of anti-HCV in the <20, [20–29], [30–39], [40–49] and >50 age groups was 0%, 0.77%, 0.92%, 1.17% and 3.12%, respectively. For those subjects, the anti-HCV prevalence increased with age (Figure 1A).

The overall prevalence of anti-HCV in 12 497 female subjects was 1.48% (185 positive), which was lower than that in the 28 772 male subjects, 1.62% (466 positive). For those subjects younger than 30 years, the prevalence of anti-HCV in females was lower than that in males, then it increased and became higher than that in males in >30 age groups (Figure 1B).

**Figure 2:**
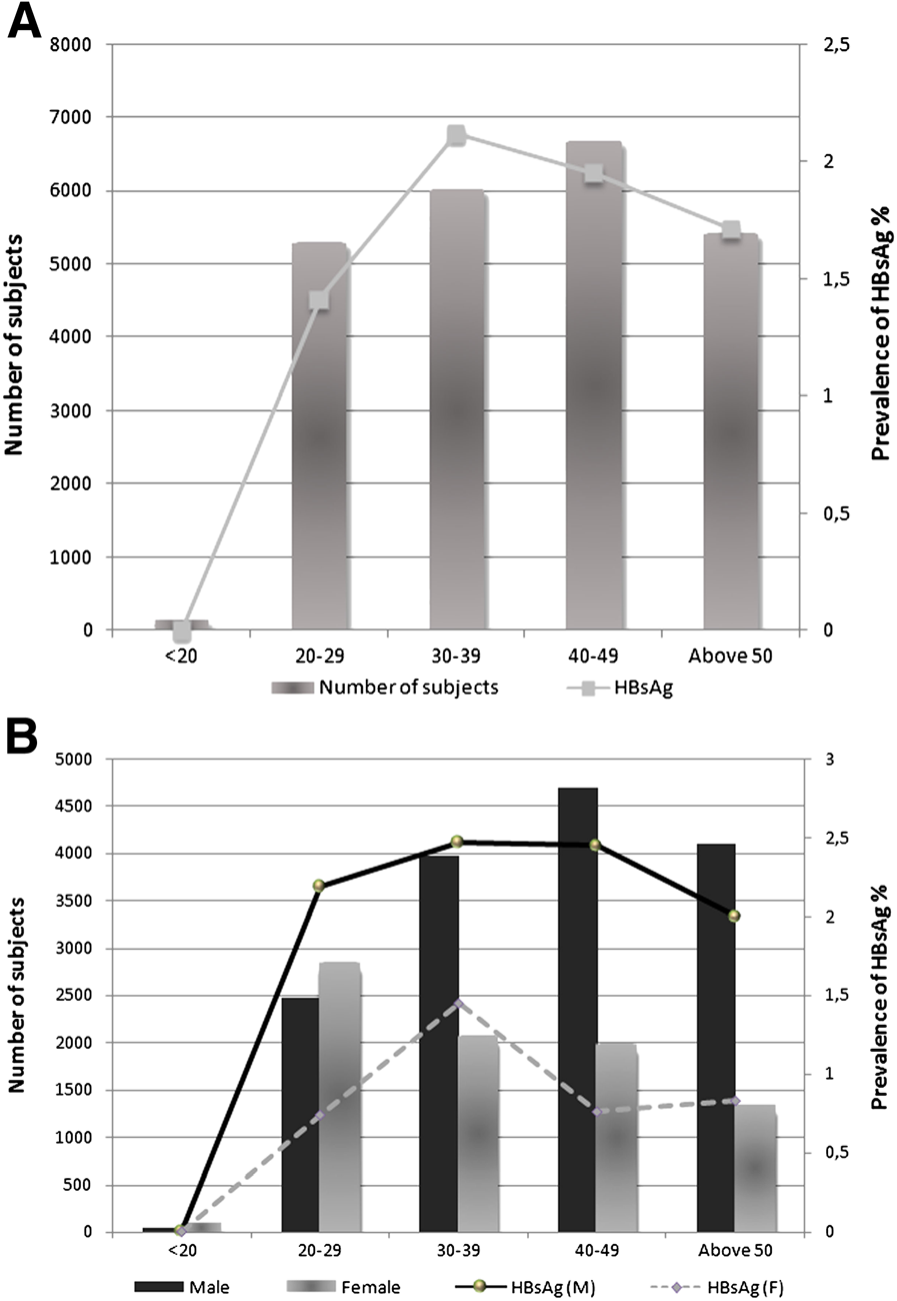
**Serological features of HBsAg prevalence in Morocco.** Bars represent the number of subjects in each group indicated by left vertical axis, and the points represent the prevalence in each group indicated by right vertical axis. The horizontal axis represents the age ranges. **A**, Overall characterization of HBsAg by age group. **B**, Overall characterization of HBsAg by gender, M, male, F, female.

The overall prevalence of HBsAg was 1.81% (426 positive) in 23578 subjects. HBsAg prevalence in <20, [20–29], [30–39], [40–49] and >50 age groups was 0%, 1.41%, 2.12%, 1.95% and 1.71%, respectively, as well as it showed an increase with age from 1.41% in [20–29] group to 2.12% and 1.95% in [30–39] and [40–49] group respectively, then decrease in the older group >50 years old to 1.71% (Figure 2A).

When the overall prevalence of HBsAg marker was analyzed according to gender, the prevalence was 2.29% and 0.93% in male and female subjects respectively. The difference was found to be statistically significant (P < 0.01), and in all groups it showed higher prevalence in males than that in females (Figure 2B). The males in age groups [30–39] and [40–49] years represent the highest seroprevalence of HBsAg found in this study (2.47% and 2.45% respectively).

Regarding the seropositivity of HBV and HCV dual infection, the presence of both HBsAg and anti-HCV antibodies was noted in 2 cases Out of 23 578 tested subjects. These two patients were male subjects who belonged to age groups 20–29 years and ≥50 years respectively.

Table 1 lists the frequency of potential risk factors reported by subjects with positive and negative anti-HCV results and the crude OR estimated by univariate analysis. Factors significantly associated with HCV infection were increasing age, dental treatment, use of glass syringes and surgical history. Conversely, no association was observed between HCV seropositivity and gender, receiving blood transfusion, tattooing, body piercing, having multiple sexual partners, acupuncture and endoscopy. On the subject of HBV infection, HBsAg positivity was significantly associated with age, male gender (OR 2.5; p < 0.01), Dental procedure history (OR 3.0; p < 0.01), Use of glass syringes (OR 2.3; p < 0.01), history of jaundice (OR 1.4; p = 0.03) and history of sexual risk behaviors (OR 2.9; p < 0.01).

**Table 1:** Distribution of Hepatitis C virus serological marker and principal risk factors in the general population December 2005-June 2011

**Risk factor**	**HCV-Positive Subjects**		**HCV-Negative Subjects**		**OR** **(CI 95%)**	**P**
	**N**	**%**	**N**	**%**		
Age						
<40	144	0.9	16 686	99.1	2.5 (2.0-2.9)	<0.01
>40	507	2.1	23 932	97.9		
Sex						
Female	185	1.5	12 312	98.5	0.9 (0.8-1.0)	0.3
Male	466	1.6	28 306	98.4		
Dental therapy						
No	244	0.8	28 351	99.2	3.8 (3.3- 4.5)	<0.01
Yes	407	3.2	12 267	96.8		
Use of glass syringes						
No	329	1.2	27 905	98.8	2.1 (1.8- 2.5)	<0.01
Yes	322	2.5	12 713	97.5		
Surgical history						
No	403	1.4	28 027	98.6	1.37 (1.2-1.6)	0.01
Yes	248	1.9	12 591	98.1		
Blood transfusion						
No	546	1.6	34 566	98.4	1.1 (0.9-1.4)	0.4
Yes	105	1.7	6052	98.3		
Piercing/tattoo						
No	599	1.6	37 694	98.4	1.1 (0.8-1.5)	0.5
Yes	52	1.7	2924	98.3		
Multiple Sexual partners						
No	634	1.6	39 806	98.4	1.3 (0.8-2.1)	0.3
Yes	17	2	812	98		
Acupuncture						
No	649	1.6	40 578	98.4	3.1 (0.7-12.9)	0.3
Yes	2	4.8	40	95.2		
Endoscopy						
No	649	1.6	40 253	98.4	0.3 (0.1-1.4)	0.2
Yes	2	0.5	365	99.5		

**OR:***Odds ratios,***CI:** confidence interval.

### Blood donors

A total of 169 605 voluntary blood donors were selected in the Transfusion Blood Center of Casablanca between January 2008 and December 2010 for the study, of whom 79.7% were males. The overall mean age was 35.7 (± 12.5) years, ranging from 18 to 65 years. The seroprevalence was estimated over the three-year study period. Among all analyzed subjects, 1057 were anti-HCV positive, representing an overall prevalence of 0.62% (Table 2). The seroprevalence was 0.57% in 2008 but subsequently increased to 0.64% in 2009 and 2010. Similarly, the prevalence of HBV was slightly increased from 1.1% in 2008 to 1.15% in 2009 and decreased to 0.63% in 2010. The overall seroprevalence rate of HBV was 0.96% corresponding to 1603 HBsAg-positive individuals.

**Table 2:** The frequency of seropositivity of HBV and HCV among blood donors at Blood Transfusion Center of Casablanca 2008-2010

**Year**	**Total screened**	**HCV positive**	**HBV positive**
	**N**	**N (%)**	**N (%)**
2008	52 279	300 (0.57)	575 (1.1)
2009	55 496	357 (0.64)	638 (1.15)
2010	61 830	400 (0.64)	390 (0.63)
Total	169 605	1057 (0.62)	1603 (0.95)

**N** = Number.

## Discussion

We report here a cross-sectional study to assess the epidemiology of HCV and HBV prevalence among the general population residing or working in the major Moroccan regions and among blood donors from the Blood Transfusion Center of Casablanca, the largest commercial and densely populated city of Morocco. To the best of our knowledge this is the largest community-based epidemiologic study of HCV and HBV infections from Morocco. The sampling procedure adopted and the high participation rate (97.3%) support the absence of selection and non-response bias, ensuring thus an accurate assessment of the prevalence in our study. Most of the previous researchers in this field were limited due to the selective nature of the survey populations. The descriptive epidemiologic data presented in this study can provide new insight into the contribution of HCV and HBV in the etiology of liver disease in Morocco.

The overall prevalence of HCV infection in the general population in our study is found to be 1.58% and was lower than the prevalence reports from close countries; Algeria (2.5%), Libya (3%) and Egypt (15%-20%) [10-12]. Our estimate was also too much lower compared to what has been reported in the prior decade by Cacoub *et al.*[13] in which the estimated HCV infection rate was 7.7%. This result is surely related to the nature of the participants enrolled in the study who were mostly hospitalized patients and those who come for consultation suffering from chronic hepatopathy or with nephrological manifestations. Most HCV-seropositive subjects in the present survey had detectable viremia (70.9%), which is consistent with findings in studies from France (80.6%) and Italy (75.9%) [14,15].

No statistically significant difference in HCV distribution according to gender was found in our study, which is similar to other reports on community-acquired hepatitis C from United states and Belgium [16,17], However, higher prevalence was observed in advanced age groups; indeed, no anti-HCV seropositive case was found in 152 subjects in age groups younger than 20 years, nevertheless the seropositivity increased progressively from adults (0.77%) to older persons (3.12%). This can be supported by two major reasons. First, before 1994, anti-HCV screening among blood donors was not conducted throughout Morocco and the association of blood transfusion with HCV seropositivity should not be surprising given that the blood product had not been previously screened for HCV. Second, the higher prevalence of HCV in older people could be attributed to a longer exposure to risk factors for HCV transmission; for instance iatrogenic transmission resulting from inadequately sterilized equipments, inappropriate reuse of supplies, etc. This risk factor is considered the main risk associated with HCV infection in the majority of participants from the general population included in this study. This same risk is the primary cause for HCV transmission in many outbreaks documented in United States and European Union health care [18,19].

Regarding HBV infection, the overall prevalence of HBsAg was significantly lower (1.81%) than that reported from Algeria (3.6%) and Tunisia (4-7%) [20,21]. Despite the large number of young in <20 group tested (152 subjects), none was positive for HBsAg, These results indicate that the strong efforts made among youngers have had some success since the HBV infant vaccination was introduced in 1992 and integrated into the national immunization program in 1999, in addition, hepatitis B immunoglobulin and HBV vaccine treatment for newborns of HBV-infected mothers at delivery could also contribute to the significant reduction of vertical or perinatal transmission of HBV.

On the other hand, the age-related prevalence of HBsAg showed a progressive decrease after 50 years (1.71%), so among participants who were older than 50 years, the prevalence of anti-HCV was higher (3.12%) than that of HBsAg (1.71%). This can be probably due to deaths caused by HBV-related cirrhosis and hepatocellular carcinoma in this group [22]. Additionally, it has been reported that HBV-associated HCC occurs approximately 10 years earlier than HCV-associated HCC [23,24]. Conversely, on the whole, the prevalence of HBV in our study population was slightly higher than that of HCV (1.81% *vs.* 1.58%). Thus, the comparative incidence of the two viruses is consistent with findings in studies conducted in India, China and Bosnia-Herzegovina [3,5,25].

Analysis by gender reveals that, the seroprevalence of hepatitis B among males is significantly higher than that found in females; this data was comparable to other reports [26], and no plausible explanation has been given for the higher rate in males in the general population but probably due to the higher exposure to occupational HBV risk factors in men, or else females clear the HBV more efficiently as compared to males [27].

Our research shows a very low prevalence (two cases among 23 578 personts tested) of hepatitis co-infection comparing with Tunisia (5%) and Egypt (22.5%) [28,29], these results indicate that the HBV positive patients investigated herein do not have an increased risk of exposure to HCV infection. Although this small sample size of reactive cases does not allow data to be compared with other reports, one Italian study found that rates of dual infection increased with age, and was more common in patients over 50 years of age [30]. In this report, the two cases were male subjects belonging to age groups 20–29 years and ≥50 years respectively. Moreover several authors have reported that HBV can reciprocally inhibit HCV replication [31]; specifically, HBV DNA replication has been shown to correlate with decreased HCV RNA levels in coinfected patients [32]. In another Italian study, coinfected patients had a rate of HCV RNA clearance of 71% compared to 14% with HCV monoinfection [31]. Furthermore, coinfected patients have been demonstrated to have lower levels of both HBV DNA and HCV RNA than corresponding mono-infected controls, inicating that concurrent suppression of both viruses by the other virus can also occur [33].

After all, according to research regarding HBV prevalence, Morocco has been estimated as a moderately endemic area; thus, in a WHO collaborative study on viral hepatitis B in which 20 countries have participated, the seroprevalence were reported was 3.3% [34]. Among barbers and their customers, the positive ratio for HBsAg was found to be 2.0% by Zahraoui *et al.* in 2004 and 28.0% by Belbacha *et al.* in 2011 [7,35]. Comparison of these findings with the HBsAg carrier rate estimated in this exhaustive investigation and our preliminary results published by Sbai *et al.*[36], shows that the epidemiological picture is changing and that the vaccination program has shifted this trend to low endemicity.

In this study, we also measured over a period of three years, the frequency of HCV and HBV infections among 169 605 voluntary blood donors assumed to be without known or obvious risk factors. 0.62% and 0.96% of subjects were tested positive for HCV antibodies and HBsAg respectively, these results were significantly lower compared with the prevalence observed among the general population reported here. This could be explained by the fact that the volunteer blood donors are a preselected healthy group based on donor questionnaires and physical examination as blood is drawn only from those applicants who appear at low risk of having blood-borne pathogens [37]. Moreover, with regard to HCV infection, this prevalence is considerably lower in comparison with that reported among blood donors in 1998 at the same institution and in 1992 at the Regional Blood Transfusion Center of Rabat (0.70% and 1.56% respectively) [38]. This observation is probably due to the improvement of the effectiveness of the medical selection through self-exclusion from blood donation and medical examination. When the results of the present study were compared with those reported from similar blood donors of other countries, a comparable prevalence of HCV antibodies has been reported in blood donors from Libya (0.69%) and Germany (0.65%) [39,40]. Studies from Tunisia (1.4 %), Senegal (0.8%) and United States (1.8%) reported higher HCV infection rate [41-43], whereas Algeria (0.18 %), Spain (0.3%), England (0.1%), Bosnia and Herzegovina (0.27%) showed lower frequency of the HCV antibodies seropositivity in comparison to the present study [20,44-46].

Measurement of HBV seropositivity, has revealed that 0.96% of the Moroccan blood donors had HBV infection. This seroprevalence is lower than that reported in Algeria (3.6%), Libya (1.28%), Turkey (1.8%) and Iran (1.07%) [20,37,39,46]. Nevertheless, it is higher than the reactivity rate reported in Rabat, Morocco (0.8%), a US community (0.15%), and Bosnia-Herzegovina (0.78%) [8,25,47].

## Conclusion

Our study throws light for the first time, on the current epidemiological status of viral hepatitis B and C infections in Morocco among the general population and blood donors. This data shows that the prevalence of HBV and HCV in blood donors was significantly lower than that observed in the general population, which emphasizes that the blood transfusion was not a contributing risk factor for transmission of neither HBV nor HCV infection. The main transmission route in Morocco is still attributed to the nosocomial exposure to contaminated instruments.

The present survey reports that the HCV infection is at an intermediate level of endemicity. It also underlines the decrease of hepatitis B incidence rate compared with previous studies. Even with a moderate prevalence, there may be a large reservoir of HCV and HBV-infected persons in Morocco, which indicates that many people are at risk of developing chronic liver disease related to viral hepatitis, added to the fact that antiviral therapy is not affordable by the vast majority of people in developing countries. Therefore, nosocomial risk prevention as well as health education among population are the main interventions that might help limiting the spread of these blood-borne infections.

## Abbreviations

HBV: Hepatitis B virus; HCV: Hepatitis C virus; HBsAg: Hepatitis B surface antigen; Anti-HCV: Anti-HCV antibody; ELISA: Enzyme-linked immunosorbent assay; RIBA: Recombinant immunoblot assay; PCR: Polymerase chain reaction; CI: Confidence Interval; OR: Odds ratio.

## Competing interests

The authors declare that they have no competing interests.

## Authors’ contributions

WB participated in data collection, carried out with FL, TPT and KE the analysis, and wrote the manuscript. AF helped in preparing Tables and Figures. ND assisted with the overall study design and supervised the statistical analysis. NN and BO have made substantial contributions to acquisition of data and their analysis. ME and AE supervised the literature review. HM and AB were responsible for the overall supervision of the study and together with SB supervised laboratory work. All authors read and approved the final manuscript.

## Pre-publication history

The pre-publication history for this paper can be accessed here:

http://www.biomedcentral.com/1471-2458/13/50/prepub
